# Gas in the Stomach and Its Treatment

**Published:** 1911-01-21

**Authors:** 


### MEDICINE.

### GAS IN THE STOMACH AND ITS TREATMENT.

FLATULENCE is a very common symptom and one which it is by no means always easy to trace back to its correct explanation in every case. It may be due to a large number of different causes, of which there are two main groups, namely: (1) air that is swallowed, and (2) gas produced within the stomach or regurgitated into the stomach from the intestine beyond. In arriving at the diagnosis it is important in the first case to be sure that the flatulence is gastric and not colonic. When there is actual belching of air there is little doubt as to the diagnosis; but in many patients it is difficult to get the air or wind up, and then doubt may arise as to whether it is really in the stomach or not. Careful and repeated observations may be required before this doubt is settled, and it may even be necessary to pass a small gastric tube to see whether the wind which produces the abnormal area of resonance in the upper regions of the abdomen can be removed thereby or not. Air swallowing is a phenomenon which accompanies even the normal act of deglutition, especially when fluids are being swallowed hastily. This accounts for the frequent development of pronounced succussion sounds in the stomach of even normal individuals after meals. In most cases no ill-effects result, but sometimes so much air may be swallowed as to cause a most uncomfortable feeling of tension across the upper part of the abdomen soon after a meal; if the patient can belch, this tension is usually relieved, but in patients who cannot belch it may produce gastric discomfort, together with a necessity for loosening belts, stays, trousers, and other garments that may constrict the upper part of the abdomen.

The differential diagnosis depends chiefly, first, upon the rapidity with which the discomfort comes on after meals; secondly, upon the time of belching; and thirdly, upon the lack of odour of putrefaction or fermentation in the gas which returns.

### Aerophagy.

There is a small group of patients in whom air is swallowed in excess by a process of periodical gulping of it down quite apart from meals. As a rule the act is at first voluntary, but it soon becomes a habit which it is very difficult to break. There has often been some temporary indigestion or other gastric disorder to begin with, and the patient has found that relief has been given to this by gulping; the air-gulping habit persists even after the dyspepsia has got better and the main symptom that remains is perpetual belching of large volumes of air at comparatively short intervals. The cure in such cases is to prevent air-swallowing. Sometimes this may be brought about voluntarily, but in many cases, in order to break the habit, it may be necessary to prevent the closure of the lips by some mechanical contrivance, such as the carrying between the teeth of a cork or other foreign body. Probably those people who can practically belch to order belong to this group of cases, although many such people are not ill at all.

### Varieties.

Fermentative or putrefactive flatulence is not by any means always of the same kind. Some authorities would like to differentiate intrinsically between fermentation and putrefaction, drawing the following distinction between the two:—

| *Fermentation.* | *Putrefaction.* |
|---|---|
| 1. Affects carbohydrates. | 1. Affects fats and proteids. |
| 2. Produces acids mainly. | 2. Produces alkalies mainly. |
| 3. Numerous bacteria, especially sarcinæ and yeasts or fungi. | 3. Colon bacilli mainly. |
| 4. Mainly in stomach or upper portion of abdomen. | 4. Mainly in large bowel or colon. |

If these points of distinction are maintained, it will be obvious that putrefaction in the stomach is far less common than is fermentation, probably occurring only when there has been long-standing obstruction of the pylorus by a healed ulcer or by new growth, or when there is intestinal obstruction, chronic or sub-acute, with regurgitation of the intestinal contents into the stomach. In either of these cases flatulence will almost certainly have been preceded by graver symptoms.

### Oral Sepsis.

It is almost a wonder that fermentative gas production within the stomach is not even commoner than it is when one thinks how general are dental caries, septic stumps, and suppurative affections in connection with the teeth and nasal cavities. The lack of gas production in many cases in which it might be expected may be ascribed to the comparative shortness of gastric digestion, the rapid removal of fermentative material by absorption, and the antiseptic action of hydrochloric acid. The action of the latter is by no means, in the strict sense of the word, antiseptic—that is to say, the strength of the hydrochloric acid is too small actually to kill offending organisms, but far greater dilution will suffice to inhibit the multiplication of microbes than is required to kill them. The standard of the bacteriologist is a lethal one, that of the physician and therapeutist merely one of inhibition.

### Treatment.

In cases that are not due to lesions requiring surgical assistance it is usually ample, when fermentative gas production is really the diagnosis, to prescribe some antiseptic of which perhaps one of the simplest is a 4 per cent. solution of borax and sodium bicarbonate. The mouth must also be attended to to minimise the rate of re-introduction of bacteria into the stomach. Charcoal, even in large doses, will not prevent gas formation, but it sometimes helps to relieve flatulence by fixing the gas. Nux vomica is an admirable remedy in many cases, especially in small but repeated doses, acting rather by accelerating the rate at which the stomach empties its contents into the duodenum than in any other way. Dietetic treatment is not to be recommended except as a temporary measure, for although the withholding of starchy foods may lessen fermentation and flatulence, the patient's general condition is apt to suffer in consequence, and the true cause of the symptom is not being tackled. In very resistant cases it may be necessary to wash out the stomach with some solution, such as weak boric acid or potassium permanganate, and in a few cases it may be temporarily necessary to stop the food by the mouth for the time being and to resort to nutrient enemata. The chief cause of failure of treatment is erroneous diagnosis. Treatment by antiseptics or by lavage is no cure for air-swallowing.

### Treatment of Functional Cases.

It may also be mentioned that some authorities hold that there is a variety of gas production in the stomach not due to fermentation but to functional errors in the nature of the secretions and undue patency of the pylorus which allows the pancreatic and intestinal juices to be regurgitated into the stomach so that the hydrochloric acid in the latter decomposes the carbonates in the secretions from lower down and gives rise to a purely chemical rather than a fermentative liberation of carbon dioxide. The patients are of nervous temperament, there is often pyrosis or waterbrash, the eructed gas bringing with it small quantities of nearly clear fluid containing little but mucus and granular débris with occasional bile pigments; auscultation of the upper part of the abdomen may detect signs of effervescence in the region of the pylorus, and the most successful treatment consists in the administration of nux vomica in association with alkalies.

